# Genital and cutaneous human papillomavirus (HPV) types in relation to conjunctival squamous cell neoplasia: A case-control study in Uganda

**DOI:** 10.1186/1750-9378-3-12

**Published:** 2008-09-10

**Authors:** Maurits NC de Koning, Keith Waddell, Joseph Magyezi, Karin Purdie, Charlotte Proby, Catherine Harwood, Sebastian Lucas, Robert Downing, Wim GV Quint, Robert Newton

**Affiliations:** 1 DDL Diagnostic Laboratory, Voorburg, The Netherlands; 2 Box 4008, Kampala, Uganda; 3 Centre for Cutaneous Research, Institute of Cell and Molecular Science, St Bartholomew's and the Royal London School of Medicine and Dentistry, Queen Mary, University of London, London E1 2AT, UK; 4 Division of Surgery and Oncology, College of Medicine, Dentistry and Nursing, University of Dundee, Ninewells Hospital, Dundee DD1 9SY, UK; 5 Dept. Histopathology, KCL School of Medicine, St. Thomas' Hospital, London, UK; 6 Centers for Disease Control and Prevention, Programme on AIDS, Uganda Virus Research Institute, PO Box 49, Entebbe, Uganda; 7 Epidemiology and Genetics Unit, Department of Health Sciences, University of York, Seebohm Rowntree Building, Heslington, York, YO10 5DD, UK

## Abstract

**Background:**

We investigated the role of infection with genital and cutaneous human papillomavirus types (HPV) in the aetiology of ocular surface squamous neoplasia (which includes both conjunctival intraepithelial neoplasia (CIN) and carcinoma) using data and biological material collected as part of a case-control study in Uganda.

**Results:**

Among 81 cases, the prevalence of genital and cutaneous HPV types in tumour tissue did not differ significantly by histological grade of the lesion. The prevalence of genital HPV types did not differ significantly between cases and controls (both 38%; Odds ratio [OR] 1.0, 95% confidence interval [CI] 0.4–2.7, p = 1.0). The prevalence of cutaneous HPV types was 22% (18/81) among cases and 3% (1/29) among controls (OR 8.0, 95% CI 1.0–169, p = 0.04).

**Conclusion:**

We find no evidence of an association between genital HPV types and ocular surface squamous neoplasia. The prevalence of cutaneous HPV was significantly higher among cases as compared to controls. Although consistent with results from two other case-control studies, the relatively low prevalence of cutaneous HPV types among cases (which does not differ by histological grade of tumour) indicates that there remains considerable uncertainty about a role for cutaneous HPV in the aetiology of this tumour.

## Background

In the years before the HIV epidemic, corneo-conjunctival intraepithelial neoplasia (CIN) and carcinoma (together called ocular surface squamous neoplasia (OSSN)) were reported to be more frequent in African countries than in Europe and the USA [1-3]. Using data from worldwide cancer registries it has been confirmed that incidence of OSSN increases markedly with proximity to the equator, presumably from increasing solar ultraviolet (UV) radiation [3]. Exposure to UV radiation is an established cause of disease. Lesions occur in sun-exposed areas of the eye [4,5], are associated with solar elastosis [4-7] and have been shown to contain classical UV-induced p53 mutations [8]. The incidence of the tumour increases with increasing levels of ambient solar radiation and associations with sun exposure and past history of skin cancer have been identified in case-control studies [3,9-11]. Additional risk factors may also be important. For example, a polymorphism of TP53 codon 72 has been linked to an increased risk of neoplasia in one study from Uganda including 107 cases and 115 controls [12]. Exposure to dust and ocular trauma have also been suggested as possible risk factors, although evidence is scant [1,13].

Since the 1980s there has been a marked increase in cases of conjunctival neoplasia, mostly in sub-Saharan Africa [14-19]. In Uganda for example, the reported incidence has more than tripled over the last decade [14,16], particularly among younger people and a link with HIV infection was suggested in case reports [20-27]. Case-control studies in several African countries [11,28-33] and cohort studies in the USA [34,35], have confirmed a roughly 10 fold excess risk of the tumour in HIV infected people compared to the uninfected; in Africa the majority of cases are HIV infected [36]. In a recent study of 414 cases in Uganda, 64% of people with conjunctival neoplasia were HIV infected and this applied to intraepithelial as well as to invasive cases [5]. The median CD4+ T lymphocyte count of HIV positive cases at diagnosis has been found in this study to be 111 cells/microL (based on results from 112 HIV infected cases) [5]. Use of antiretroviral therapy has been shown to cause tumour regression in an otherwise inoperable case [37]. A recent report from the USA did not find strong associations with level of immunosuppression in HIV infected people, but the study included only 15 cases of the disease [35]. An excess risk has also been reported among immunosuppressed cancer patients and organ transplant recipients (although the number of cases remains small) [38-42].

However, the clear excess risk of ocular surface epithelial dysplasias among HIV infected people (and among immunosuppressed renal transplant recipients) suggests a role for an underlying infection in the aetiology [43,44]. Although an active search for other new oncogenic infections is ongoing, no new candidate virus (if one exists) has yet been identified [45]. A causal relationship between persistent infection with several (high risk) genital human papillomavirus (HPV) types and cancer of the uterine cervix is established. In non-melanoma skin carcinogenesis, a role has been suggested for cutaneous HPV types from the *betapapillomavirus *genus. A variety of HPV types has already been identified in some, but not in all, tumour specimens from several small case series and results from case-control studies have, to date, been inconclusive [2]. Here we present results on the association of genital and cutaneous (from the *betapapillomavirus *genus) HPV types in relation to ocular surface epithelial neoplasias from a case-control study in Uganda, together with a review of published evidence.

## Results

Paraffin-embedded biopsy material was available for 81 cases (mean age 35 years) and for 29 controls (mean age 30 years). Among cases, 17 people had lesions graded as CIN (conjunctival intraepithelial neplasia) I, 18 were CIN II, 22 were CIN III and 24 people had an invasive carcinoma. Fifty two cases (64%) were HIV seropositive, 22 (27%) were seronegative and the HIV serostatus was unknown for seven people (9%). Among controls, 15 people had pinguecula, 3 had chronic inflammation, two had a pyogenic granuloma, two had a cavernous angioma and seven had a variety of other diagnoses. Ten controls (34%) were HIV seropositive and 19 (66%) were seronegative (Table 1). There were significantly more HIV seropositive cases than controls (70% (52/74; seven cases had unknown serostatus) versus 34%; p < 0.001).

**Table 1:** Distribution of cases and controls by age, sex and HIV serostatus

	**Percentage of cases (n) n = 81**	**Percentage of controls (n) n = 29**
**Age**		
**15–28**	27% (22)	38% (11)
**29–32**	27% (22)	31% (9)
**33–70**	46% (37)	31% (9)

**Sex**		
**Male**	44% (36)	48% (14)
**Female**	56% (45)	52% (15)

**HIV serostatus**		
**Negative**	27% (22)	66% (19)
**Positive**	64% (52)	34% (10)
**Unknown**	9% (7)	0% (0)

Table 2 shows the prevalence of genital and cutaneous HPV among controls, stratified by age, sex and HIV serostatus – none of the apparent differences was statistically significant (at the 5% level). Table 3 shows the proportion of cases with evidence of genital or cutaneous HPV DNA in tumour tissue, stratified by the histological grade of the lesion (CIN I – III and invasive carcinoma). The prevalence of genital and cutaneous HPV types did not differ significantly by histological grade of the lesion, but at all grades, the prevalence of genital HPV types was higher than that of cutaneous types. For all tumour grades combined, this difference was statistically significant (38% versus 22%; p = 0.03).

**Table 2:** The proportion of controls with evidence of infection with HPV, stratified by age, sex and HIV serostatus

	**All HPV**	**Genital HPV**	**Cutaneous-HPV**
**Age**			
**15–28**	27% (3/11)	27% (3/11)	9% (1/11)
**29–32**	33% (3/9)	33% (3/9)	0% (0/9)
**33–70**	56% (5/9)	56% (5/9)	0% (0/9)

**Sex**			
**Male**	29% (4/14)	29% (4/14)	0% (0/14)
**Female**	47% (7/15)	47% (7/15)	7% (1/15)

**HIV serostatus**			
**Negative**	32% (6/19)	32% (6/19)	0% (0/19)
**Positive**	50% (5/10)	50% (5/10)	10% (1/10)

**TOTAL**	38% (11/29)	38% (11/29)	3% (1/29)

None of the apparent differences in prevalence of HPV by age, sex or HIV sero-status was statistically significant.

**Table 3:** The proportion of cases with evidence of infection with HPV, stratified by histological grade of tumour

	**All HPV**	**Genital HPV**	**Cutaneous-HPV**^1^
**CIN I**	47% (8/17)	35% (6/17)	29% (5/17)
**CIN II**	56% (10/18)	50% (9/18)	28% (5/18)
**CIN III**	45% (10/22)	27% (6/22)	23% (5/22)
**Invasive**	42% (10/24)	42% (10/24)	13% (3/24)
			
**TOTAL**	47% (38/81)	38% (31/81)	22% (18/81)

1. χ^2 ^(trend) = 1.9; p = 0.2

Overall, the prevalence of genital HPV types did not differ between cases and controls (38% [31/81] in cases and 38% [11/29] among controls; OR 1.0, 95% CI 0.4–2.7, p = 1.0). The genital HPV types identified were 6, 11, 16, 18, 31, 33, 35, 44, 51, 52, 66 and two that were unclassifiable. High risk genital types were identified in 13 cases (types 16 [eight people], 51 and 66 [in one person], 18, 35, 51 and 52 [one person each]) and in three controls (types 31 and 33 [two people]). The most frequently detected genital type was HPV 11, which was found in 22 cases and in 10 controls. The prevalence of cutaneous HPV types was 22% (18/81) among cases and 3% (1/29) among controls (OR 8.0, 95%CI 1.0–168.5, p = 0.04) and did not differ significantly between HIV infected and uninfected cases and controls (Table 4). The cutaneous HPV types identified were 5, 8, 14, 17, 19, 23, 36, 37, 80, plus 9 that were unclassifiable; HPV 14 was identified in three cases and types 8, 17 and 23 were found in two people each. Evidence of infection with more than one HPV type was identified in tissue from 19 cases and four controls.

**Table 4:** The proportion of cases and controls with evidence of infection with HPV, stratified by HIV serostatus

	**All HPV**^1^			**Genital HPV**^2^			**Cutaneous-HPV**^3^		
	
	**Case**^4^	**Control**	**Odds Ratio (95% CI)**	**Case**^4^	**Control**	**Odds Ratio (95% CI)**	**Case**^4^	**Control**	**Odds Ratio (95% CI)**
**HIV seronegative**	45% (10/22)	32% (6/19)	1.8 (0.4–7.9)	36% (8/22)	32% (6/19)	1.2 (0.3–5.5)	27% (6/22)	0% (0/19)	∞ (1.8–∞)
**HIV seropositive**	48% (25/52)	50% (5/10)	0.9 (0.2–4.3)	40% (21/52)	50% (5/10)	0.8 (0.2–3.8)	21% (11/52)	10% (1/10)	2.2 (0.2–52)
**TOTAL**	47% (38/81)	38% (11/29)	1.5 (0.6–3.8)	38% (31/81)	38% (11/29)	1.0 (0.4–2.7)	22% (18/81)	3% (1/29)	8.0 (1.0–169)

1. More than one HPV type was identified in tissue from 19 cases and four controls

2. Genital HPV types investigated: 6, 11, 16, 18, 31, 33–35, 39, 40, 42–45, 51–54, 56, 58, 59, 66, 68, 70, 74. Genital HPV types identified: 6, 11, 16, 18, 31, 33, 35, 44, 51, 52, 66, plus two unclassifiable; high risk genital types were identified in 13 cases (types 16 [eight people], 51 and 66 [in one person] 18, 35, 51 and 52 [one person each]) and in three controls (types 31 and 33 [two people]); HPV 11 was most frequently detected (22 cases and 10 controls)

3. Cutaneous HPV types investigated: 5, 8, 9, 12, 14, 15, 17, 19–25, 36–38, 47, 49, 75, 76, 80, 92, 93, 96. Cutaneous HPV types identified: 5, 8, 14, 17, 19, 23, 36, 37, 80, plus 9 unclassifiable; HPV 14 was identified in three cases and types 8, 17 and 23 were found in two people each

4. Among cases, 7 had unknown HIV serostatus

## Discussion

Our findings demonstrate that both genital and cutaneous HPVs can be found in conjunctival tissue – the genital types were more frequently identified. However, we found no evidence that genital types were associated with ocular surface squamous neoplasia. In relation to cutaneous HPV, results reported here are broadly consistent with those from two other studies – the prevalence was significantly higher among cases than among controls. However, the prevalence of cutaneous HPV was still relatively low among cases and did not differ by histological grade of the lesion.

A comprehensive review of the published literature identified 12 case reports or case series in which the prevalence of HPV in tumour tissue from patients with ocular surface squamous neoplasia was investigated [46-57]. Eleven studies tested for HPV 16; seven also looked for evidence of infection with HPV 18; four studies also included HPV 6 and/or 11, one looked at HPV 2 and in one study the specific genital HPV type was not specified. Only three studies included more than 20 cases, the largest having 38. The prevalence of detectable HPV varied from 0% to 93% (summarised in Table 5) – much of this variation might be explained by the differing laboratory methodologies employed across individual studies. Sixteen case-control studies were identified and are summarised in Table 6, together with results from this investigation [7,11,30,58-70]. With the exception of one study, in which HPV type was not specified, all of the studies investigated HPV 16, nine also investigated HPV 18 and 5 investigated HPV 45. There is considerable heterogeneity in results. For example, in relation to HPV 16, four studies demonstrated a positive association and eleven showed no association with ocular surface squamous neoplasia (five studies failed to identify HPV 16 in either the cases or controls). In most studies, type-specific methods of HPV detection were used and so the types shown in the tables were the only ones that were tested for.

**Table 5:** Summary of case series investigating the prevalence of HPV DNA in tumour tissue from patients with ocular surface squamous neoplasias.

**Study [Reference]**	**Detection method**	**Number HPV positive/total (%)**	**HPV type**
McDonnell *et al*, 1987 [46]	*In situ *hybridisation (ISH)	0/28 (0%)	HPV 2, 6, 16, 18
McDonnell *et al*, 1989 [47]	PCR	1/1 (100%)	HPV 16
Lauer *et al*, 1990 [48]	PCR	4/5 (80%)	HPV 16
		2/5 (40%)	HPV18
Odrich *et al*, 1991 [49]	PCR	2/2 (100%)	HPV 16
McDonnell *et a*l, 1992 [50]	PCR	33/38 (87%)	HPV 16
Tuppurainen *et al*, 1992 [51]	ISH and PCR	0/4 (0%)	HPV 6, 11, 16 and 18
Serna *et al*, 1995 [52]	PCR	1/9 (11%)	HPV 16
Nakamura *et al*, 1997 [53]	ISH and PCR	2/8 (25%)	HPV 16
		2/8 (25%)	HPV 18
Toth *et al*, 2000 [54]	PCR	5/23 (9%)	HPV types not specified
Eng *et al*, 2002 [55]	PCR	0/20 (0%)	HPV 6, 11, 16, 18
Moubayed *et al*, 2004 [56]	ISH	12/14 (86%)	HPV 16
		13/14 (93%)	HPV 18
		12/14 (86%)	HPV 6 and 11
Reszec and Sulkowski, 2005 [57]	PCR	1/11 (9%)	HPV 16
		1/11 (9%)	HPV 18

**Table 6:** Summary of case-control studies investigating various HPV types in the aetiology of ocular surface squamous neoplasias

**Study**	**Detection method**	**Number HPV positive/total (%)**		**HPV type**
		**Case**	**Control**	
McDonnell *et al*, 1986 [58]	*In situ *HPV antigen detection	5/61 (8%)	0/6 (0%)	Unknown genital HPV type
McDonnell *et al*, 1989 [59]	PCR	6/6 (100%)	0/6 (0%)	HPV 16
Saegusa *et al*, 1995 [60]	ISH and PCR	3/8 (38%)	0/12 (0%)	HPV 16
Adachi *et al*, 1995 [61]	PCR	1/5 (20%)	0/9 (0%)	HPV 16
Waddell *et al*, 1996 [30]	PCR	7/20 (35%)	2/15 (13%)	HPV 16
Karcioglu and Issa, 1997 [62]	PCR	4/45 (9%)	8/70 (11%)	HPV 16
		10/45 (22%)	10/70 (14%)	HPV 18
Tabrizi *et al*, 1997 [63]	PCR	20/88 (23%)	5/66 (8%)	HPV 16 or 18
Dushku *et al*, 1999 [64]	PCR	0/8 (0%)	0/16 (0%)	L1 (all types)
Palazzi *et al*, 2000 [65]	PCR	2/30 (7%)	1/30 (3%)	HPV 16
Scott *et al*, 2002 [66]	ISH and *in situ *reverse transcriptase PCR			
		5/10 (50%)	0/5 (0%)	HPV 16
		5/10 (50%)	0/5 (0%)	HPV 18
Newton *et al*, 2002 [11]	Serological analysis	8/39 (21%)	43/418 (10%)	HPV 16
		4/39 (10%)	16/418 (4%)	HPV 18
		2/39 (5%)	24/418 (6%)	HPV 45
Tulvatana et al, 2003 [7]	PCR	0/28 (0%)	0/23 (0%)	Multiple types
Waddell *et al*, 2003 [67]	Serological analysis	37/253 (15%)	6/37 (16)	HPV 16
Ateenyi-Agaba *et al*, 2004 [68]	PCR	0/21 (0%)	0/22 (0%)	HPV 16, 18 and 45
		0/22 (0%)	2/22 (9%)	HPV 11
		18/21 (86%)	7/20 (35%)	Multiple cutaneous HPV types
Tornesello *et al*, 2006 [69]	PCR	0/86 (0%)	1/63 (2%)	HPV 6
		2/86 (2%)	0/63 (0%)	HPV 18
		15/86 (17%)	0/63 (0%)	Multiple cutaneous HPV types
Sen *et al*, 2007 [70]	In situ HPV antigen detection	0/30 (0%)	0/30 (0%)	Multiple genital HPV types
de Koning *et al *[this study]	PCR	31/81 (38%)	11/29 (38%)	Multiple genital HPV types
		18/81 (22%)	1/29 (3%)	Multiple cutaneous HPV types^1^

Only three studies (including this one [68,69]) investigated cutaneous HPV types – each demonstrated a significantly higher prevalence of cutaneous HPV in cases as compared to controls (summarised in Table 7). Two of the three studies examined the prevalence according to histological grade of tumour (this study and reference 69) and no association was demonstrated in either.

**Table 7:** Summary of case-control studies investigating cutaneous HPV types in the aetiology of ocular surface squamous neoplasias

**Study [Reference]**	**Prevalence of cutaneous HPV (number/total)**		**Odds Ratio (95% Confidence Interval) and p value**
		
	**Cases**	**Controls**	
Ateenyi-Agaba et al, 2004 [68]	86% (18/21)	35% (7/20)	12.0 (1.7–84.9), p = 0.002^1^
Tornesello et al, 2006 [69]	17% (15/86)	0% (0/63)	∞ (2.5–∞), p = 0.001^2^
de Koning [this study]	22% (18/81)	3% (1/29)	8.0 (1.0–168.5), p = 0.04

1. For comparative purposes, the unadjusted odds ratio is shown

2. Estimated using Fisher exact test

There is substantial variation in HPV prevalence rates between different studies, which may have arisen, in part, because of differences in patient selection, sample taking, preparation and storage and detection method. Even for PCR as a detection system, there are many variables that influence the sensitivity and specificity and so could impact on the reported prevalence. These include PCR design (nested, broad spectrum or type-specific), the size of the amplified product and the choice of the polymerase used. This review was not done to draw attention to these differences, but rather to show that there is no consistent evidence for a causal association between HPV and OSSN. In addition, however, it should be noted that the total number of cases and controls studied in this and in other studies, remains relatively small.

Results reported here are also broadly similar to those from case-control studies investigating the role of HPV in the aetiology of cutaneous squamous cell carcinoma (SCC) [71,72]. Moderate associations between cutaneous HPV types and cutaneous SCC have been identified, but doubt remains about whether this is causal. It has been suggested that the increased serorecognition of HPV among cases as compared to controls may arise as a result of tumour formation [73]. Some support for this view comes from a recent small prospective study, in which the seroprevalence of antibodies against the L1 antigen of 38 HPV types among 39 cases of cutaneous squamous cell carcinoma (SCC) for whom plasma was collected prior to diagnosis (incident) and 80 controls was examined [72]. Fifteen cases having already developed SCC at blood collection (prevalent) were also tested. There were no statistically significant differences in the seroprevalence of antibodies against any of the HPV types examined between incident cases and controls, nor was there a difference in the seroprevalence of multiple infections. However, consistent with results from published case-control studies, the seroprevalence against many cutaneous HPV types was higher among prevalent cases than among either incident cases or controls. This might suggest that if HPV is involved in the aetiology of cutaneous squamous carcinoma, the process occurs close to the time of diagnosis, or that the antibody response observed in people with the tumour is a consequence of tumour formation.

The possibility that the presence of a tumour facilitates detection of antibodies against HPV is supported by the findings of Favre et al (2000), who reported a higher seroprevalence of HPV-5 among patients with burns or with proliferative cutaneous autoimmune diseases than among controls [74]. Patients with psoriasis, involving abnormal keratinocyte differentiation and proliferation, have also shown a high HPV-5 seroprevalence [75]. This is thought to arise as a consequence of cell proliferation in the skin providing an environment that favours viral replication, resulting in a rise in antibodies against the relevant HPV type. Similarly, there is debate concerning the results obtained from studies using tests for cutaneous HPV DNA. The prevalence of HPV DNA was significantly lower in tumour biopsies than in swabs of the tested lesion [76]. Furthermore, evidence of cutaneous HPV DNA has been found to be both highly prevalent and persistent in the healthy population [77]. It is possible that the results reported here reflect a similar situation. However, there is now some preliminary evidence from studies of molecular mechanisms, suggesting that HPV might interact with ultra-violet radiation disturbing apoptotic pathways and leading to cell immortalization [78]. Transforming properties of E6 and E7 proteins of some cutaneous HPV types have also been described (reviewed in reference [71]). It remains to be established what role, if any, HPV plays in the pathological processes that lead to the development of both conjunctival and cutaneous squamous cell neoplasia.

It should be noted that the relatively high percentage of samples with unclassified cutaneous HPV types could represent infections with novel types of which only subgenomic amplicons have been sequenced [79]. However, the other possibility is that these were infections with low copy numbers of one of the 25 tested cutaneous HPV types allowing only for general detection and not the identification of specific types. With the broad spectrum SPF_10 _PCR – DEIA (see Methods section) more than 50 HPV types can be detected. It cannot, therefore, be excluded that the two cases with an indeterminate genital HPV result actually represent a cutaneous HPV type. The SPF_10_-LiPA_25 _system amplifies a small fragment from 65 base pairs and is therefore very suitable for the testing of paraffin-embedded, formalin-fixed samples. Although the conjunctiva represent mucosal tissue, the detection of genital HPV types in 40% of the HIV seronegative cases and in 32% of the HIV seronegative controls was unexpected. This finding indicates that the natural history of HPV and their tissue tropism is not fully understood.

## Conclusion

We find no evidence of an association between genital HPV types and ocular surface squamous neoplasia. The prevalence of cutaneous HPV was significantly higher among cases as compared to controls. Although consistent with results from two other case-control studies, the relatively low prevalence of cutaneous HPV types among cases (which does not differ by histological grade of tumour) indicates that there remains considerable uncertainty about a role for cutaneous HPV in the aetiology of this tumour.

## Methods

### Participants

From November 1995 to May 2001 in country-wide clinics, anyone with a suspect corneo-conjunctival lesion was offered removal and histology, and enrolment in a follow-up study with home visits. HIV serology was also offered after pre-test counselling. Lesions were photographed and details of the eyes and general health were recorded and analysed in EPI INFO version 6. Those who subsequently turned out to have lesions other than ocular surface squamous neoplasia were used as a control group in the analyses of HPV.

### Consent and ethical approval

Information about the disease, its treatment and HIV testing was given in private in vernacular by counsellors, and consent confirmed by signature or thumbprint. The study was approved by the Science and Ethics Committee of the Uganda Virus Research Institute, and by the Uganda National Council for Science and Technology.

### Serology and histopathology

Venous blood was taken and screening tests for HIV antibodies done, with confirmation at the Uganda Virus Research Institute (two enzyme immunoassay tests in parallel, with Western blot if required). Biopsies went to St Thomas' Hospital London for histopathology. CIN was classified (by SBL) into 3 stages according to one, two or three thirds thickness being dysplastic; invasive tumours were diagnosed when the epithelial basement membrane was breached.

### HPV typing

HPV analyses were performed on DNA isolated from formalin-fixed, paraffin-embedded specimens. Chances of contamination during the cutting of the sections were minimised by discarding the initial section that was cut to remove any environmental contamination which had occurred while blocks were stored and by changing cryostat blades in between sections. DNA was extracted from the sections in a cabinet which had been UV-treated to remove any contaminating DNA. Additionally, 15 negative DNA isolation controls were included. For both the genital HPV test and the beta HPV test, 10 μl of a 20 ng/μl DNA solution per specimen was used as input for the PCR analyses. Genital HPV genotyping was carried out using the SPF_10_-LiPA_25 _system (SPF_10 _HPV LiPA, version 1; manufactured by Labo Bio-Medical Products, Rijswijk, The Netherlands) as described previously [80,81]. Briefly, the broad spectrum SPF_10 _PCR amplifies a 65-base pair fragment from the L1 region of the HPV genome. By using biotinylated reverse primers the amplimers could be captured onto streptavidin-coated microtiter plates. After denaturation of the PCR products by alkaline treatment, a defined cocktail of digoxigenin-labeled probes was used to detect HPV positive samples. This method that is designated the HPV DNA Enzyme Immunoassay (DEIA) provides an optical density value and is able to detect more than 50 HPV types [82]. Amplimers from positive samples were used for subsequent genotyping of twenty-five individual genital HPV genotypes (high-risk HPV: 16, 18, 31, 33, 35, 39, 45, 51, 52, 56, 58, 59, 66, 68, 70, and low-risk HPV: 6, 11, 34, 40, 42–44, 53, 54, 74) simultaneously in a reverse hybridisation assay (RHA). Beta HPV genotyping was performed with the PM-PCR RHA method (The skin (beta) HPV prototype research assay; Diassay BV, Rijswijk, The Netherlands) [83]. It consists of a broad spectrum PCR specific for the amplification of the betaPV genus and targets a fragment of 117 bp from the E1 region of the HPV genome. Combined with the RHA, it was possible to identify 25 beta HPV types (i.e., HPV type 5, 8, 9, 12, 14, 15, 17, 19–25, 36–38, 47, 49, 75, 76, 80, 92, 93 and 96). As no DEIA was developed for this assay all amplimers were directly analysed by RHA.

### Review methods

Case series and controlled studies of HPV and ocular surface squamous neoplasia published up to April 2008, were identified through a medline search [1966–2006; search terms (exploded, all subheadings): squamous cell carcinoma, human papillomavirus (HPV), conjunctival cancer], supplemented by searches of references in identified papers, by hand searches of relevant journals and by direct contact with authors. No restriction was placed on language of publication. No attempt was made to identify unpublished studies or to obtain unpublished data from published studies. There were no prospective studies. The odds ratios used here are either those presented in the paper or, where none were provided, they were estimated for each study by the authors, using published figures.

## Competing interests

The authors declare that they have no competing interests.

## Authors' contributions

KW, JM, RD and RN conducted the original study and collected all the biological material used for work described here. MK and WQ developed the HPV assays, which MK used in this study, with assistance from KP, CP and CH. SL conducted the histopathology. RN conducted the statistical analyses. The manuscript was drafted by RN and MK. All authors read, contributed to and approved the manuscript.

## References

[B1] Templeton AC, Templeton AC (1973). Tumours of the eye and adnexa. Tumours of a Tropical Country: A survey of Uganda 1964–1968 Recent Result Cancer Research.

[B2] Newton R (1996). A review of the aetiology of squamous cell carcinoma of the conjunctiva. Br J Cancer.

[B3] Newton R, Ferlay J, Reeves G, Beral V, Parkin DM (1996). Incidence of squamous cell carcinoma of the eye increases with increasing levels of ambient solar ultraviolet radiation. Lancet.

[B4] McKelvie PA, Daniell M, McNab A, Loughnan M, Santamaria JD (2002). Squamous cell carcinoma of the conjunctiva: a series of 26 cases. Br J Ophthalmol.

[B5] Waddell K, Downing R, Lucas S, Newton R (2006). Corneo-conjunctival carcinoma associated with human immunodeficiency virus type-1 (HIV-1) in Uganda. Eye.

[B6] Clear A, Chirambo M, Hutt M (1979). Solar keratosis, pterygium, and squamous cell carcinoma of the conjunctiva in Malawi. Br J Ophthalmol.

[B7] Tulvatana W, Bhattarakosol P, Sansopha L, Sipiyarak W, Kowitdamrong E, Paisuntornsug T, Karnsawai S (2003). Risk factors for conjunctival squamous cell neoplasia: a matched case-control study. Br J Ophthalmol.

[B8] Ateenyi-Agaba C, Dai M, Le Calvez F, Katongole-Mbidde E, Smet A, Tommasino M, Franceschi S, Hainaut P, Weiderpass E (2004). TP53 mutations in squamous-cell carcinomas of the conjunctiva: evidence for UV-induced mutagenesis. Mutagenesis.

[B9] Sun EC, Fears TR, Goedert JJ (1997). Epidemiology of squamous cell conjunctival cancer. Cancer Epidemiology, Biomarkers & Prevention.

[B10] Lee GA, Williams G, Hirst LW, Green AC (1994). Risk Factors in the Development of Ocular Surface Epithelial Dysplasia. Ophthalmology.

[B11] Newton R, Ziegler J, Ateenyi-Agaba C, Bousarghin L, Casabonne D, Beral V, Mbidde E, Carpenter L, Reeves G, Parkin DM, Wabinga H, Mbulaiteye S, Jaffe H, Bourboulia D, Boshoff C, Coursaget P, the Uganda Kaposi's Sarcoma Study Group (2002). The epidemiology of conjunctival squamous cell carcinoma in Uganda. Br J Cancer.

[B12] Tornesello ML, Waddell KM, Duraturo ML, Biryahwaho B, Downing R, Lucas SB, Giani U, Buonaguro L, Buonaguro FM (2005). TP53 codon 72 polymorphism and risk of conjunctival squamous cell carcinoma in Uganda. Cancer Detection and Prevention.

[B13] Margo CE, Groden LR (1986). Squamous cell carcinoma of the cornea and conjunctiva following a thermal burn of the eye. Cornea.

[B14] Parkin DM, Wabinga H, Nambooze S, Wabwire-Mangen F (1999). AIDS-related cancers in Africa: maturation of the epidemic in Uganda. AIDS.

[B15] Poole TRG (1999). Conjunctival squamous cell carcinoma in Tanzania. Br J Ophthalmol.

[B16] Wabinga H, Parkin D, Wabwire-Mangen F, Nambooze S (2000). Trends in cancer incidence in Kyadondo county, Uganda, 1960–1997. Br J Cancer.

[B17] Pola EC, Masanganise R, Rusakaniko S (2003). The trend of ocular surface squamous neoplasia among ocular surface tumour biopsies submitted for histology from Sekuru Kaguvi Eye Unit, Harare between 1996 and 2000. Cent Afr J Med.

[B18] Kalua K (2007). Treatment of conjunctival intraepithelial neoplasia in Africa. Br J Ophthalmol (electronic letters; 22^nd ^January in response to Waddell and Newton. Br J Ophthalmol.

[B19] Waddell KM, Newton R (2007). The aetiology and associations of conjunctival intraepithelial neoplasia – further evidence. Br J Ophthalmol.

[B20] Winward KE, Curtin VT (1989). Conjunctival squamous cell carcinoma in a patient with human immunodeficiency virus infection. Am J Ophthalmol.

[B21] Kim RY, Seiff SR, Howes EL, O'Donnell JJ (1990). Necrotizing scleritis secondary to conjunctival squamous cell carcinoma in aquired immunodeficiency syndrome. Am J Ophthalmol.

[B22] Denis P, Charpentier D, Roudier M (1994). Conjunctival epidermoid carcinoma and human immunodeficiency virus. J Fr Ophthalmol.

[B23] Mahomed A, Chetty R (2002). Human immunodeficiency virus infection, Bcl-2, p53 protein and Ki-67 analysis in ocular surface squamous neoplasia. Arch Ophthalmol.

[B24] Wilhelm F, Herz E, McArthur C, Werschnik C (2004). HIV seroprevalence in ophthalmologic patients of Cameroon. Ophthalmologe.

[B25] Chinogurei TS, Masanganise R, Rusakaniko S, Sibanda E (2006). Ocular surface squamous neoplasia (OSSN) and human immunodeficiency virus at Sekuru Kaguvi Eye Unit in Zimbabwe: the role of operational research studies in a resource poor environment?. Cent Afr J Med.

[B26] Chisi SK, Kollmann MK, Karimurio J (2006). Conjunctival squamous cell carcinoma in patients with human immunodeficiency virus infection seen at two hospitals in Kenya. East Afr Med J.

[B27] Osahon AI, Onunu AN (2007). Ocular disorders in patients infected with the human immunodeficiency virus at the University of Benin Teaching Hospital, Benin City, Nigeria. Niger J Clin Pract.

[B28] Kestelyn P, Stevens A, Ndayambaje, Hanssens M, Perre P van de (1990). HIV and conjunctival malignancies. Lancet.

[B29] Ateenyi-Agaba C (1995). Conjunctival squamous-cell carcinoma associated with HIV infection in Kampala, Uganda. Lancet.

[B30] Waddell K, Lewallen S, Lucas S, Atenyi-Agaba C, Herrington C, Liomba G (1996). Carcinoma of the conjunctiva and HIV infection in Uganda and Malawi. Br J Ophthalmol.

[B31] Newton R, Ziegler J, Beral V, Mbidde E, Carpenter L, Wabinga H, Mbulataiye S, Appleby P, Reeves G, Jaffe H, the Uganda Kaposi's Sarcoma Study Group (2001). A case control study of Human Immunodeficiency Virus infection and cancer in adults and children residing in Kampala, Uganda. Int J Cancer.

[B32] Porges Y, Groisman GM (2003). Prevalence of HIV in conjunctival squamous cell neoplasia in an African provincial hospital. Cornea.

[B33] Timm A, Stropahl G, Schittowski M, Sinzidi C, Kayembe D, Guthoff R (2004). Association of malignant tumors of the conjunctiva and HIV infection in Kinshasa (D.R. Congo). First results. Ophthalmologe.

[B34] Goedert JJ, Cote TR (1995). Conjunctival malignant disease with AIDS in USA. Lancet.

[B35] Guech-Ongey M, Engels EA, Goedert JJ, Biggar RJ, Mbulaiteye SM (2008). Elivated risk for squamous cell carcinoma of the conjunctiva among adults with AIDS in the United States. Int J Cancer.

[B36] Ateenyi-Agaba C, Newton R (1999). Squamous cell carcinoma of the conjunctiva: an HIV-associated cancer. Bulletin of the Royal Society of Tropical Health.

[B37] Holkar S, Mudhar HS, Jain A, Gupta M, Rogstad KE, Parsons MA, Singh AD, Rennie IG (2005). Regression of invasive conjunctival squamous carcinoma in an HIV-positive patient on antiretroviral therapy. Int J STD AIDS.

[B38] Kushner FH, Mushen RL (1975). Conjunctival squamous cell carcinoma combined with malignant lymphoma. Am J Ophthalmol.

[B39] Macarez R, Bossis S, Robinet A, Le Callonnec A, Charlin JF, Colin J (1999). Conjunctival epithelial neoplasias in organ transplant patients receiving cyclosporine therapy. Cornea.

[B40] Shelil AE, Shields CL, Shields JA, Eagle RC (2003). Aggressive conjunctival squamous cell carcinoma in a patient following liver transplantation. Arch Ophthalmol.

[B41] Pournaras JA, Chamot L, Uffer S, Zografos L (2007). Conjunctival intraepithelial neoplasia in a patient treated with tacrolimus after liver transplantation. Cornea.

[B42] Vajdic CM, van Leeuwen MT, McDonald SP, McCredie MR, Law M, Chapman JR, Webster AC, Kaldor JM, Grulich AE (2007). Increased incidence of squamous cell carcinoma of the eye after kidney transplantation. J Natl Cancer Inst.

[B43] Beral V, Newton R (1998). Overview of the epidemiology of immunodeficiency associated cancers. Monogr Natl Cancer Inst.

[B44] Newton R, Beral V, Weiss R, Newton R, Beral V, Weiss R (1999). Human Immunodeficiency Virus Infection and Cancer. Cancer Surveys, Infections and Human cancer.

[B45] Feng H, Taylor JL, Benos PV, Newton R, Waddell K, Lucas SB, Chang Y, Moore PS (2007). Human transcriptome subtraction using short sequence tags to search for tumor viruses. J Virol.

[B46] McDonnell PJ, McDonnell JM, Kessis T, Green WR, Shah KV (1987). Detection of human papillomavirus type 6/11 DNA in conjunctival papillomas by *in situ *hybridization with radioactive probes. Hum Pathol.

[B47] McDonnell JM, McDonnell PJ, Stout WC, Martin WJ (1989). Human papillomavirus DNA in a recurrent squamous carcinoma of the eyelid. Arch Ophthalmol.

[B48] Lauer SA, Malter JS, Meier JR (1990). Human papillomavirus type 18 in conjunctival intraepithelial neoplasia. Am J Ophthalmol.

[B49] Odrich MG, Jakobiec FA, Lancaster WD, Kenyon KR, Kelly LD, Kornmehl EW, Steinert RF, Grove AS, Shore JW, Gregoire L, Albert DM (1991). A spectrum of bilateral squamous conjunctival tumors associated with human papillomavirus type 16. Ophthalmology.

[B50] McDonnell JM, McDonnell PJ, Sun YY (1992). Human papillomavirus DNA in tissues and ocular surface swabs of patients with conjunctival epithelial neoplasia. Invest Ophthalmol Vis Sci.

[B51] Tuppurainen K, Raninen A, Kosunen O, Kankkunen JP, Kellokoski J, Syrjanen S, Mantyjarvi M, Syrjanen K (1992). Squamous cell carcinoma of the conjunctiva: Failure to demonstrate HPV DNA by in situ hybridization and polymerase chain reaction. Acta Ophthalmol (Copenh).

[B52] Serna A, Corredor JC, Benavides J, Ureta J, Orozco O (1995). Human papillomavirus (HPV) and squamous cell carcinoma of the conjunctiva. Neoplasia.

[B53] Nakamura Y, Mashima Y, Kameyama K, Mukai M, Oguchi Y (1997). Detection of human papillomavirus infection in squamous tumours of the conjunctiva and lacrimal sac by immunohistochemistry, *in situ *hybridisation and polymerase chain reaction. Br J Ophthalmol.

[B54] Toth J, Karcioglu ZA, Moshfeghi AA, Issa TM, Al-Ma'ani JR, Patel KV (2000). The relationship between human papillomavirus and p53 gene in conjunctival squamous cell carcinoma. Cornea.

[B55] Eng HL, Lin TM, Chen SY, Wu SM, Chen WJ (2002). Failure to detect human papillomavirus DNA in malignant epithelial neoplasms of conjunctiva by polymerase chain reaction. Am J Clin Pathol.

[B56] Moubayed P, Mwakyoma H, Schneider DT (2004). High frequency of human papillomavirus 6/11, 16 and 18 infections in precancerous lesions and squamous cell carcinoma of the conjunctiva in subtropical Tanzania. Am J Clin Pathol.

[B57] Reszec J, Sulkowski S (2005). The expression of P53 protein and infection of human papillomavirus in conjunctival and eyelid neoplasms. Int J Mol Med.

[B58] McDonnell JM, McDonnell PJ, Mounts P, Wu T-C, Green WR (1986). Demonstration of papillomavirus capsid antigen in human conjunctival neoplasia. Arch Ophthalmol.

[B59] McDonnell JM, Mayr AJ, Martin WJ (1989). DNA of Human Papillomavirus type 16 in dysplastic and malignant lesions of the conjunctiva and cornea. N Engl J Med.

[B60] Saegusa M, Takano Y, Hashimura M, Okayasu I, Shiga J (1995). HPV type 16 in conjunctival and junctional papilloma, dysplasia and squamous cell carcinoma. J Clin Pathol.

[B61] Adachi W, Nishida K, Shimizu A, Soma H, Yokoi N, Kinoshita S (1995). Human papillomavirus in the conjunctiva in ocular surface diseases. Jpn J Clin Ophthalmol.

[B62] Karcioglu ZA, Issa TM (1997). Human papillomavirus in neoplastic and non-neoplastic conditions of the external eye. Br J Ophthalmol.

[B63] Tabrizi SN, McCurrach FE, Drewe RH, Borg AJ, Garland SM, Taylor HR (1997). Human papillomavirus in corneal and conjunctival carcinoma. Aust N Z J Ophthalmol.

[B64] Dushku N, Hatcher SL, Albert DM, Reid TW (1999). P53 expression and relation to human papillomavirus infection in pingueculae, pterygia and limbal tumours. Arch Ophthalmol.

[B65] Palazzi MA, Erwenne CM, Villa LL (2000). Detection of human papillomavirus in epithelial lesions of the conjunctiva. Sao Paulo Med J.

[B66] Scott IU, Karp CL, Nuovo GJ (2002). Human papillomavirus 16 and 18 expression in conjunctival intraepithelial neoplasia. Ophthalmology.

[B67] Waddell K, Magyezi J, Bousarghin L, Coursaget P, Lucas S, Downing R, Casabonne D, Newton R (2003). Antibodies against human papillomavirus type 16 (HPV-16) and conjunctival squamous cell neoplasia in Uganda. Br J Cancer.

[B68] Ateenyi-Agaba C, Weiderpass E, Smet A, Dong W, Dai M, Kahwa B, Wabinga H, Katongole-Mbidde E, Franceschi S, Tommasino M (2004). *Epidermodysplasia verruciformis *human papillomavirus types and carcinoma of the conjunctiva: a pilot study. Br J Cancer.

[B69] Tornesello ML, Duraturo ML, Waddell KM, Biryahwaho B, Downing R, Balinandi S, Lucas SB, Buonaguro L, Buonaguro FM (2006). Evaluating the role of human papillomaviruses in conjunctival neoplasia. Br J Cancer.

[B70] Sen S, Sharma A, Panda A (2007). Immunohistochemical localisation of human papillomavirus in conjunctival neoplasias: a retrospective study. Indian J Ophthalmol.

[B71] Nindl I, Gottschling M, Stockfleth E (2007). Human papillomaviruses and non-melanoma skin cancer: basic virology and clinical manifestations. Dis Markers.

[B72] Casabonne D, Michael K, Waterboer T, Pawlita M, Forslund O, Burk RD, Travis R, Key T, Newton R (2007). A prospective pilot study of antibodies against human papillomavirus (HPV) and cutaneous squamous cell carcinoma (SCC) nested in the Oxford component of the European Prospective Investigation into Cancer and Nutrition (EPIC-Oxford). Int J Cancer.

[B73] Feltkamp MC, Broer R, di Summa FM, Struijk L, Meijden E van der, Verlaan BP, Westendorp RG, ter Schegget J, Spaan WJ, Bouwes Bavinck JN (2003). Seroreactivity to epidermodysplasia verruciformis-related human papillomavirus types is associated with non-melanoma skin cancer. Cancer Res.

[B74] Favre M, Majewski S, Noszczyk B, Maienfisch F, Pura A, Orth G, Jablonska S (2000). Antibodies to human papillomavirus type 5 are generated in epidermal repair processes. J Invest Dermatol.

[B75] Favre M, Orth G, Majewski S, Baloul S, Pura A, Jablonska S (1998). Psoriasis: A possible reservoir for human papillomavirus type 5, the virus associated with skin carcinomas of epidermodysplasia verruciformis. J Invest Dermatol.

[B76] Forslund O, Lindelof B, Hradil E, Nordin P, Stenquist B, Kirnbauer R, Slupetzky K, Dillner J (2004). High prevalence of cutaneous human papillomavirus DNA on the top off skin tumors but not in "stripped" biopsies from the same samples. J Invest Dermatol.

[B77] de Koning MN, Struijk L, Bavinck JN, Kleter B, ter Schegget J, Quint WG, Feltkamp MC (2007). Betapapillomaviruses frequently persist in the skin of healthy individuals. J Gen Virol.

[B78] Akgul B, Cooke JC, Storey A (2006). HPV-associated skin disease. J Pathol.

[B79] Forslund O, Iftner T, Andersson K, Lindelof B, Hradil E, Nordin P, Stenquist B, Kirnbauer R, Dillner J, de Villiers EM, Viraskin Study Group (2007). Cutaneous human papillomaviruses found in sun-exposed skin: beta-papillomavirus species 2 predominates in squamous cell carcinoma. J Infect Dis.

[B80] Kleter B, van Doorn LJ, Ter Schegget J (1998). Novel short-fragment PCR assay for highly sensitive broad-spectrum detection of anogenital human papillomaviruses. Am J Pathol.

[B81] Kleter B, van Doorn LJ, Schrauwen L (1999). Development and clinical evaluation of a highly sensitive PCR-reverse hybridization line probe assay for detection and identification of anogenital human papillomavirus. J Clin Microbiol.

[B82] van Doorn LJ, Molijn A, Kleter B, Quint WGV, Colau B (2006). Highly effective detection of human papillomavirus 16 and 18 DNA by a testing algorithm combining broad-spectrum and type-specific PCR. J Clin Microbiol.

[B83] de Koning MNC, Quint WGV, Struijk L (2006). Evaluation of a Novel Highly Sensitive, Broad-Spectrum PCR-Reverse Hybridization Assay for Detection and Identification of Beta-Papillomavirus DNA. J Clin Microbiol.

